# Preparation, Anticoagulant and Antioxidant Properties of Glucosamine-Heparin Salt

**DOI:** 10.3390/md20100646

**Published:** 2022-10-18

**Authors:** Qin Miao, Qing Li, Wenqiang Tan, Yingqi Mi, Bing Ma, Jingjing Zhang, Zhanyong Guo

**Affiliations:** 1 Key Laboratory of Coastal Biology and Bioresource Utilization, Yantai Institute of Coastal Zone Research, Chinese Academy of Sciences, Yantai 264003, China; 2 Center for Ocean Mega-Science, Chinese Academy of Sciences, 7 Nanhai Road, Qingdao 266071, China; 3 University of Chinese Academy of Sciences, Beijing 100049, China

**Keywords:** glucosamine-heparin salt, glucosamine hydrochloride, heparin sodium, antioxidant activity, anticoagulant activity

## Abstract

Excessive inorganic ions in vivo may lead to electrolyte disorders and induce damage to the human body. Therefore, preparation of enhanced bioactivity compounds, composed of activated organic cations and organic anions, is of great interest among researchers. In this work, glucosamine-heparin salt (GHS) was primarily synthesized with positively charged glucosamine hydrochloride (GAH) and negatively charged heparin sodium (Heps) by ion exchange method. Then, the detailed structural information of the GHS was characterized by FTIR, ^1^H NMR spectroscopy and ICP-MS. In addition, its anticoagulant potency and antioxidant properties were evaluated, respectively. The results demonstrated that GHS salt achieved enhanced antioxidant activities, including 98.78% of O_2_•^−^ radical scavenging activity, 91.23% of •OH radical scavenging rate and 66.49% of DPPH radical scavenging capacity at 1.6 mg/mL, severally. Meanwhile, anticoagulant potency (ATTP) of GHS strengthened from 153.10 ± 17.14 to 180.03 ± 6.02 at 0.75 μmol/L. Thus, introducing cationic glucosamine residues into GHS could improve its anticoagulant activity. The findings suggest that GHS product with a small amount of inorganic ions can greatly abate the prime cost of antioxidants and anticoagulants, and has significant economic benefits and practical significance.

## 1. Introduction

Glucosamine (GlcN), 2-amino-2-deoxyalpha-d-glucose, is a naturally occurring amino monosaccharide, which is composed of a glucose molecule attached to an amino group [1,2,3]. GlcN intensively occurs in gastric mucosalinings, ligaments, cartilage, joints, connective tissues and synovial liquid, and it is an essential biochemical precursor component for the synthesis of glycolipids, glycosaminoglycan chains, proteoglycans and all amino sugars in human body [4,5,6]. Generally, glucosamine could be produced commercially by extraction of chitin from shells of crustaceans followed by several methods including enzymatic hydrolysis, chemical hydrolysis and microbial production [1,4]. In recent decades, there has been a significant pharmacological increase in the use of GlcN to relieve symptoms of osteoarthritis (OA) and it has not demonstrated any prominent toxicity and adverse effects for humans [7,8]. In addition, many investigations have demonstrated that GlcN possesses various pharmacological and biological functions in anti-aging, antifibrotic, neuroprotective, cardioprotective, anticancer, antineoplastic, antioxidative, bacteriostatic, anti-inflammatory and immunostimulating [5,9].

As one of the members of glycosaminoglycans, heparin (Hep) is a linear polymer with prominent alternating residues of (1-4)-glycosidically linked α-d-glucosamine and uronic acid (β-d-glucuronic acid or α-l-iduronic acid) [10,11,12,13]. Many studies have reported that the molecular structures of heparin and its derivatives are intricate and difficult to characterize, due to the variety of sulfation degree and its distribution [14,15]. The heparin chain is often 6-*O*-sulfated, 3-*O*-sulfated and *N*-sulfated on the d-glucosamine residues as well as 2-*O*-sulfated on uronic acid residues [16,17,18]. Therefore, heparin is a heterogeneous and polyanionic polysaccharide [14,19]. Because of the intricate structure and polydispersity of Hep, its biological properties are significantly affected [20]. In recent decades, numerous pharmacological and biological activities of Hep, low molecular weight heparins (LMWHs) and its derivatives, such as anti-microbial, anti-tumor, anti-inflammatory, anti-viral, anti-lipidemic, regulating angiogenesis and anti-fibrotic activities were found in comprehensive studies [21,22,23,24,25].

Anticoagulants are commonly used to treat life-threatening conditions in medical operations, and Hep as an antithrombotic and anticoagulant drug has been clinically employed for over 80 years [26,27,28]. Meanwhile, numerous clinical reports have evidenced that reactive oxygen species (ROS), such as hydroxyl radicals, superoxide anion and peroxyl, can induce neurodegenerative diseases, and glucosamine hydrochloride (GAH) has been proven to have better free radical scavenging activities [1,29]. However, Hep, LMWHs and GAH contain inorganic ions, such as sodium ions (Na^+^), calcium ions (Ca^2+^) in heparin molecules and chloride ions (Cl^−^) in glucosamine hydrochloride. Those inorganic ions mainly improve the stability of compounds, without special biological activities. Additionally, it may lead to an electrolyte disorder of the human body once excessive inorganic ions are in vivo. Therefore, a series of significative methods should be proposed urgently to enhance the activity of ionic groups and prevent the harm of overdose with inorganic salt ions to the human body.

Until now, some common commercial products, such as glucosamine hydrochloride (GAH), glucosamine sulfate (GAS), heparin sodium, heparin calcium and their physical mixtures, have been documented and applied extensively [8,30]. Nevertheless, the study of combining the positively charged glucosamine salt (GAH or GAS) and negatively charged heparin salt (heparin sodium or heparin calcium) to form a single pure glucosamine organic salt compound has not been investigated. In this paper, in order to obtain a kind of glucosamine organic salt compound with small amount of inorganic ions, negatively charged heparin sodium (Heps) was combined with positively charged glucosamine hydrochloride to synthesize glucosamine-heparin salt (GHS) by ion exchange method. Then, the detailed structural information of the GHS was characterized using FTIR, ^1^H NMR spectroscopy and ICP-MS. Subsequently, its anticoagulant activity and antioxidant properties against hydroxyl radical, DPPH radical and superoxide radical in vitro were investigated, respectively.

## 2. Results and Discussion

### 2.1. Characterization of Glucosamine-Heparin Salt

The structural information of raw samples and products was characterized with Fourier transform infrared (FTIR), ^1^H Nuclear magnetic resonance (^1^H NMR), inductively coupled plasma mass spectrometer (ICP-MS) and titration-precipitation with silver nitrate.

#### 2.1.1. FTIR Spectra

**Figure A1:**
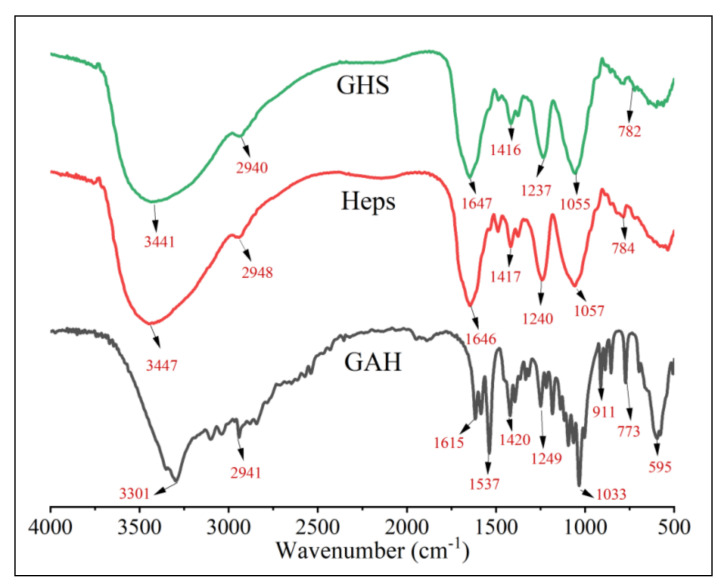
FTIR spectra of GAH, Heps and GHS.

The FTIR spectra of GAH, Heps and GHS were displayed in Figure A1 of Appendix A. In Figure A1 of Appendix A, for GAH, the major characteristic absorptions appeared at 3301 cm^−1^ (the –NH and –OH vibrations), 2941 cm^−1^ (the C–H vibration), 1615 cm^−^^1^ and 1080 cm^−^^1^ (the C–O stretching vibration) [8]. For Heps, the characteristic absorption bands were 784 cm^−1^ and 3447 cm^−1^ (–OH and –NH stretching vibrations), and the absorption bands appeared at 2948 cm^−1^ (the C–H stretching vibration), 1646 cm^−1^ (the C=O stretching vibration of uronic acid), 1240 cm^−1^ (the S=O stretching vibration), 1417 cm^−1^ (the COO– vibration), 1057 cm^−1^ (the C–O–C vibration) and 750~800 cm^−1^ (the C–O–S stretching vibration of aminohexose sulfate group), respectively [18,31]. For GHS, the major characteristic absorptions appeared at 3441 cm^−1^, 2940 cm^−1^, 1647 cm^−1^, 1416 cm^−1^, 1237 cm^−1^, 1055 cm^−1^ and 782 cm^−1^.

No new characteristic absorptions appeared when comparing the FTIR spectra of the new GHS derivative and that of Heps. Hence, the FTIR spectra information could not accurately characterize the GHS and its structural identification was further analyzed by ^1^H NMR.

#### 2.1.2. ^1^H NMR Spectra

**Figure 1:**
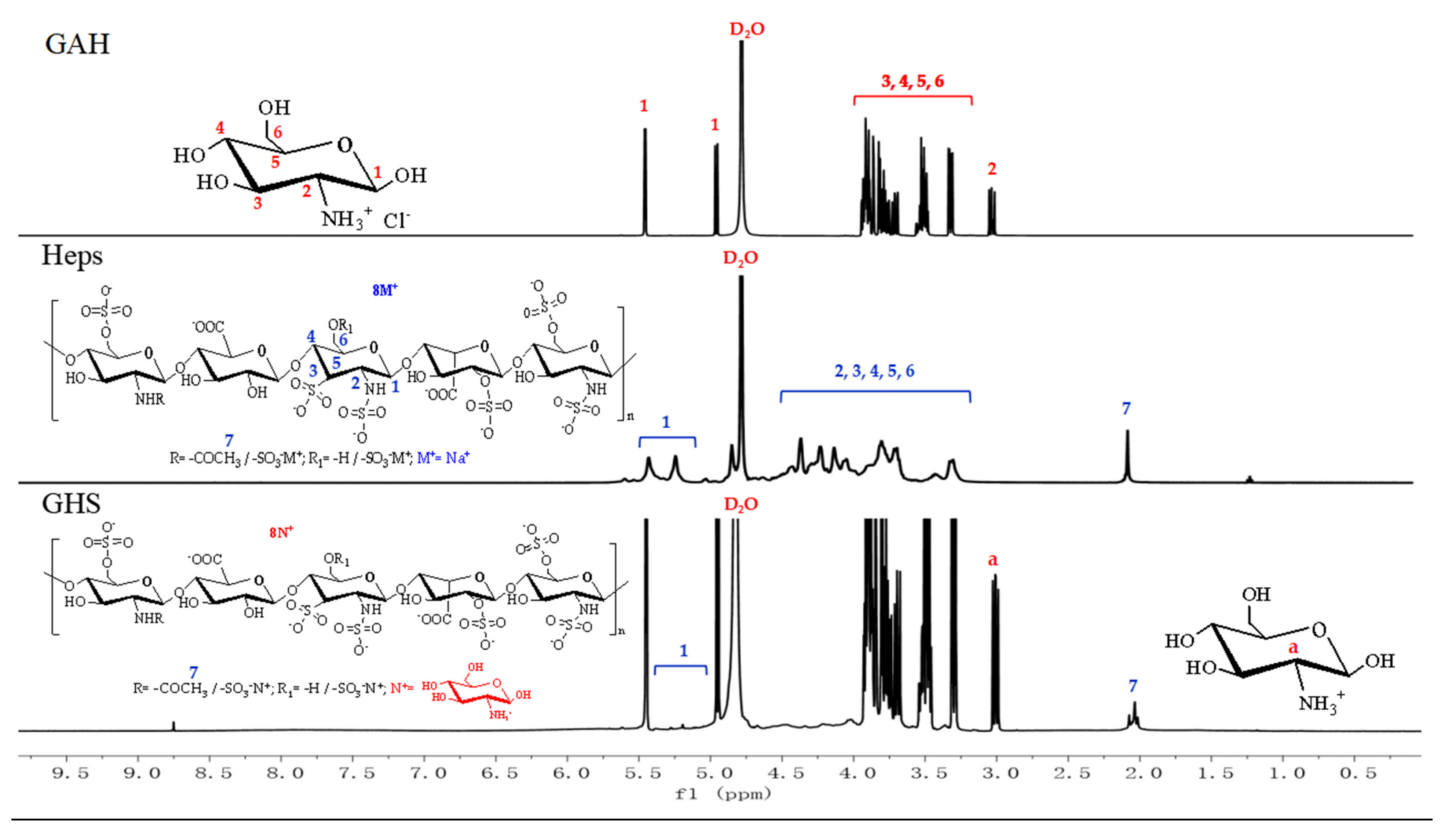
^1^H NMR spectra of GAH, Heps and GHS. ^a^ C-2 hydrogen atom of the exchanged cationic GlcN.

The ^1^H NMR spectra of GAH, Heps and GHS are shown in Figure 1.

In Figure 1, the ^1^H NMR spectra of GAH showed that the peaks at 5.44 ppm and 4.91 ppm represented the hydrogens of 1 position equatorial bond and vertical bond on the sugar ring of the glucosamine molecule. Several peaks in the range of 3.91~3.30 ppm and 3.00 ppm were assigned to the hydrogens at the 3, 4, 5, 6 positions and 2 position of the glucosamine ring [8]. ^1^H NMR spectra of Heps displayed that the peaks between 5.41 ppm and 4.99 ppm belonged to the hydrogens of 1 position anomeric carbons on the sugar rings of glucosamine, l-iduloside, *N*-acetyl glucosamine and d-glucuronic acid. The peaks in the range of 4.40~3.32 ppm were the hydrogens of 2, 3, 4, 5 positions on the sugar rings of glucosamine, l-iduloside, *N*-acetyl glucosamine, d-glucuronic acid and 6 position on the hexatomic ring of *N*-acetyl glucosamine. The signature peak representing *N*-acetylated group was shown at 2.05 ppm [28,32,33]. Concurrently, from the ^1^H NMR spectra of GHS, the poignant hydrogen peaks of 1 position, 2 position and 3, 4, 5, 6 positions on the sugar ring of GAH glucosamine appeared at 5.45 ppm, 4.94 ppm, 3.00 ppm and 3.93~3.30 ppm. The peaks in the range of 5.42~5.00 ppm were the hydrogens of 1 positions on the sugar rings of glucosamine, l-iduloside, *N*-acetylglucosamine and d-glucuronic acid of Heps. The broad absorption peaks of hydrogens of 2, 3, 4, 5 positions on the Heps sugar rings of glucosamine, l-iduloside, *N*-acetyl glucosamine, d-glucuronic acid and 6 position on the hexatomic ring of *N*-acetyl glucosamine appeared in the range of 4.39~3.35 ppm. Additionally, the signature peak on behalf of *N*-acetylated group appeared at 2.03 ppm. In other words, all of the symbolic hydrogen spectrum peaks of GAH and Heps concurrently occurred in the ^1^H NMR spectra of GHS. Consequently, it demonstrated that the GHS product was more likely to be prepared successfully.

#### 2.1.3. ICP-MS and Titration-Precipitation with Silver Nitrate

**Figure 2:**
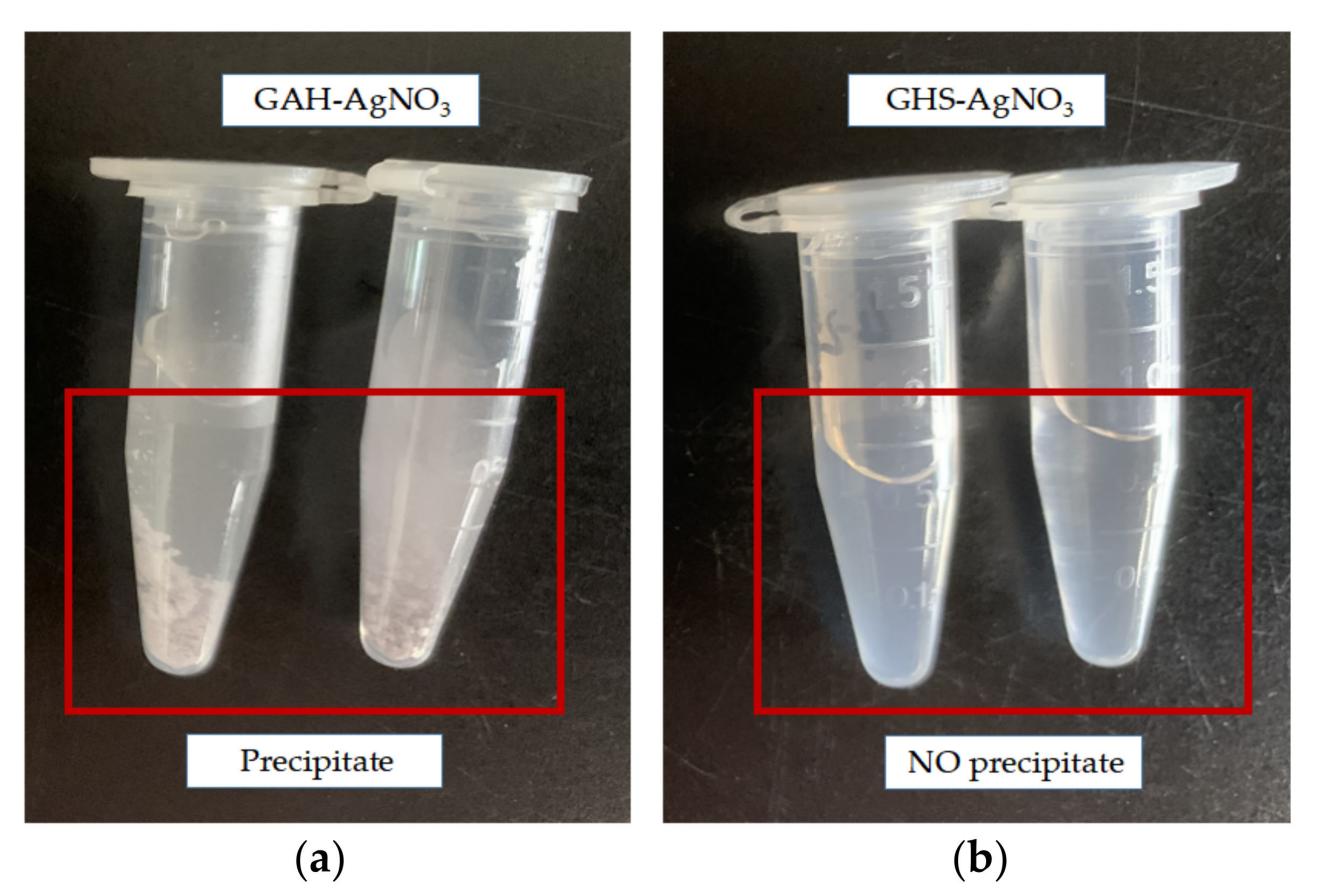
Determination of chloride ion (Cl^−^) of GHS and GAH: (**a**) mixture of GAH and AgNO_3_; (**b**) mixture of GHS and AgNO_3_.

In order to further characterize the synthetic GHS product and ensure the accuracy of preparation, experiments concerning determination of sodium ions (Na^+^) and chloride ions (Cl^−^) contained in sample compounds were carried out. Na^+^ ions contained in Heps and GHS were assessed and determined by inductively coupled plasma mass spectrometer (ICP-MS), meanwhile Cl^−^ ions in GHS and GAH were detected and evaluated by the titration-precipitation method with silver nitrate as the titrant. The detailed results are shown in Table 1 and Figure 2.

From Table 1, we can see that sodium ion (Na^+^) content of Heps (2.4 × 10^5^ ± 1.10) was significantly higher than that of GHS (4520 ± 0.50) at the same sample concentration of 1.0 mg/mL. In other words, content of Na^+^ ions in synthetic GHS product, 50 times lower than that of Heps, was significantly decreased after dialysis with the dialysis bags (Mw = 500). Furthermore, in Figure 2, precipitate appeared once silver nitrate (AgNO_3_) was added to GAH, whereas no sediment occurred when GHS was mixed with AgNO_3_. Hence, GAH possessed plentiful chloride ions while Cl^−^ ions barely consisted of GHS product, suggesting that Cl^−^ had been practically completely exchanged and eliminated by the ion exchange method. Thereby, after dialysis with the dialysis bags (Mw = 500), the negatively charged Heps with a large molecular weight and large amount of positively charged GAH were retained to form GHS by electrovalent bond according to the charge balance principle of compound. Meanwhile, inorganic ions of Na^+^ and Cl^−^ were dialyzed and removed. In summary, we were sufficiently convinced that GHS was successfully synthesized.

### 2.2. Yield and Degree of Substitution (DS)

**Figure 3:**
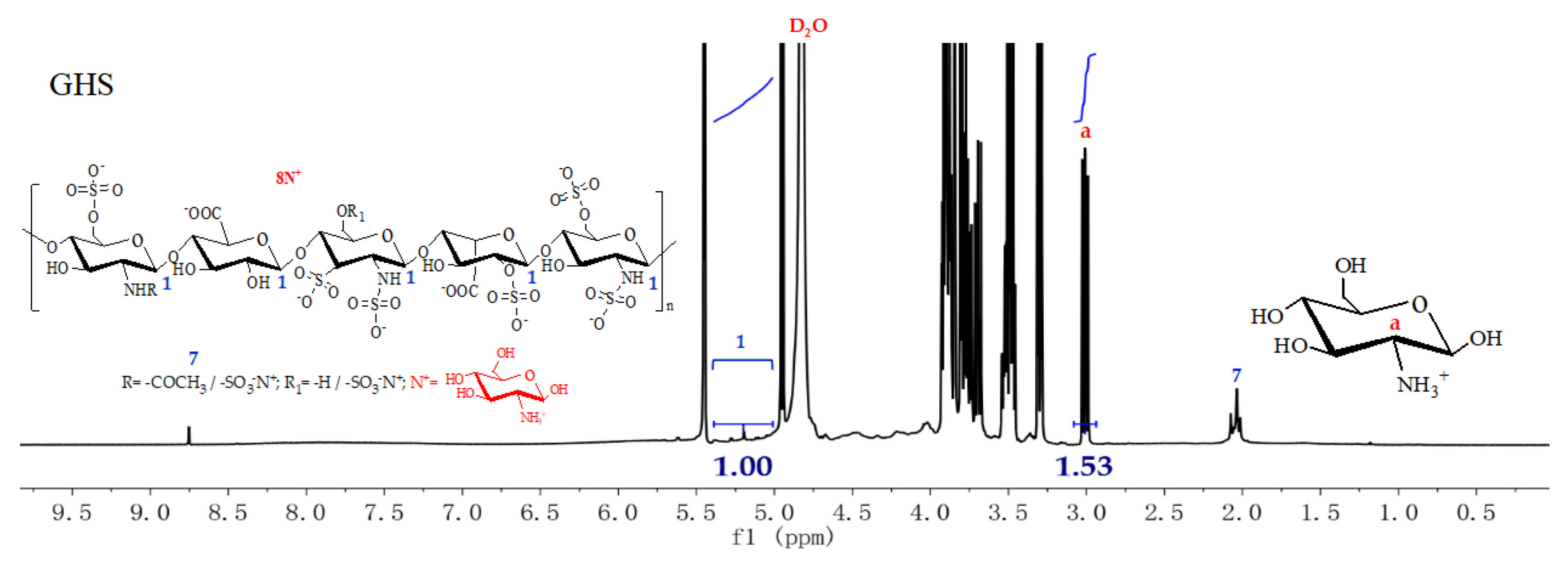
^1^H NMR spectrum of GHS and its integral values of hydrogen protons. ^a^ C-2 hydrogen atom of the exchanged cationic GlcN.

The yield of GHS product was achieved on the basis of the ratio (%) of the actual weight (g) to the theoretical weight (g). By calculation, the yield of GHS was 73.45%. Meanwhile, the DS of GHS product was quantitatively calculated by ratio of integral of the hydrogen atom of the C-1-position of Heps sugar rings to the carbon 2 bonded hydrogen atom of exchanged positively charged GlcN with some coefficients. According to the ^1^H NMR spectra, the carbon 1 bonded hydrogen atom of Heps appearing at 5.42~5.00 ppm was used as the integral standard peak and the detailed DS of GHS was calculated according to the following Equation (1). The ^1^H NMR spectrum of GHS and its integral values of hydrogen protons is shown in Figure 3. Then, according to Figure 3 and Equation (1), the DS of GHS was obtained, which was 95.63%.

DS(%) = (5 × I_Ha, GHS_)/(8 × I_H1, GHS_) × 100 (1)

where I_Ha, GHS_ was the integral of C-2 hydrogen atom of the exchanged cationic GlcN (3.00 ppm) of GHS, I_H1, GHS_ was the integral of H_1_ of the Heps backbone (5.42~5.00 ppm), 5 represented the number of sugar rings in one repeating unit and 8 represented the number of anion groups in one repeating unit.

### 2.3. Anticoagulant Ability Analysis

The anticoagulant activities of samples in vitro were measured by activated partial thromboplastin times (APTTs), prothrombin times (PTs) and thrombin times (TTs) assays using Heps as the positive control group. Several APTTs, PTs and TTs of normal saline, GAH, Heps and GHS were determined. The detailed anticoagulant results are displayed in Table 2.

From Table 2, some rules could be summarized as follows. On the one hand, the values of APTT, PT and TT of GAH were super-approximated to that of normal saline, manifesting that GAH itself had almost no anticoagulant activity. It was also consistent with the previous reports [7,9]. While, compared with the saline control groups, the anticoagulant activities (APTTs, PTs and TTs) of Heps and GHS were prolonged in a concentration-dependent manner. For example, the TTs indexes of GHS were 22.90 ± 0.36, 28.70 ± 1.31 and ≥100 s when the sample concentrations were at 0.75, 1.50 and 2.25 μmol/L, respectively. On the other hand, in the positive control, GHS emerged as prolonging APTTs, PTs and TTs compared to that of heparin sodium. It had been reported that the prolongation of APTTs, PTs and TTs suggested inhibition of the intrinsic coagulation pathway [34]. In other words, the anticoagulant capacity of GHS was enhanced and primarily ascribed to the incorporation of glucosamine residues, which enriched the pentasaccharide sequences of GHS and played an essential role in activating and binding antithrombin III (AT) [16,35]. This might be because that incorporation of cationic glucosamine residues promoted the hydrophobicity of heparin sodium which was beneficial for increasing the anticoagulant activity [24,34,36]. In addition, it was not difficult to discover that the anticoagulant activity of GHS was improved slightly compared with that of Heps. However, the prepared GHS compound, which was composed of synergistic actions (activated glucosamine cations and heparin anions) possessed inviting preponderances, such as a comparatively high DS, good yield rate, low preparation cost and small amount of inorganic ions. Accordingly, the findings suggested that GHS has a tremendous potential to be investigated as a kind of outstanding anticoagulant.

### 2.4. Antioxidant Activity Analysis

**Figure 4:**
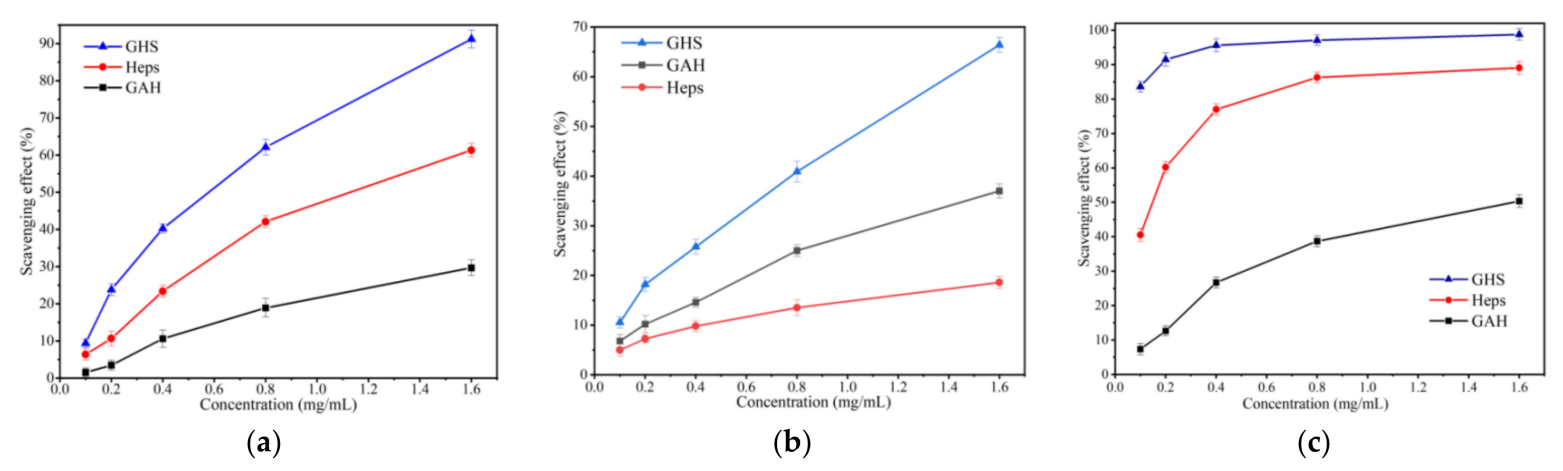
(**a**) Hydroxyl radical scavenging activity; (**b**) DPPH radical scavenging ability and (**c**) superoxide radical scavenging activity of GAH, Heps and GHS.

Antioxidant activities of GHS, GAH and Heps were tested by adopting the scavenging capacity assay on the hydroxyl radical, DPPH radical and superoxide radical. Additionally, the detailed scavenging effect results against •OH radical, DPPH radical and O_2_•^−^ radical are displayed in Figure 4.

#### 2.4.1. Hydroxyl Radical Scavenging Activity

Data in Figure 4a introduced the determination results of hydroxyl radical scavenging ability of glucosamine hydrochloride, heparin sodium, and glucosamine-heparin salt at different concentrations. In this experiment, the samples concentration measurement range was from 0.1 to 1.6 mg/mL and the scavenging ability against hydroxyl radicals showed positive correlation with the sample concentration. In addition, the results manifested that the hydroxyl radical removal rate of the GHS was better than the original GAH and Heps. It is worth mentioning that the hydroxyl radical scavenging rate of GHS achieved 91.23% compared to 61.35% scavenging rate of Heps and 29.68% of GAH, when the concentration was 1.6 mg/mL. That is, GHS demonstrated higher hydroxyl radical removal capacity than GAH and Heps.

#### 2.4.2. DPPH Radical Scavenging Ability

Evaluation of DPPH radical scavenging activities of GAH, Hep and GHS at different concentrations is shown in Figure 4b. As demonstrated in the figure, the DPPH radical scavenging ability was concentration-dependent for all the tested samples and the scavenging effect against the DPPH radical of GAH did not change significantly as the concentration increased from 0.1 to 1.6 mg/mL. Moreover, we could find that scavenging rates of GHS of 10.55%, 18.23%, 25.77%, 40.97% and 66.49% were constantly higher than those of GAH and Heps. It suggested that GHS had a great influence on the DPPH radical scavenging activity.

#### 2.4.3. Superoxide Radical Scavenging Ability

Similar rules can be found in Figure 4c. Briefly, the obvious decreasing trend in the superoxide anion radical scavenging effect was found for GHS, Heps and GAH with the decreased scavenging abilities of 83.63%, 40.55% and 7.36% at 0.1 mg/mL; 97.12%, 77.01% and 26.73% at 0.4 mg/mL. The superoxide radical scavenging indexes of GHS were 98.78%, 97.12%, 95.67%, 91.51%, and 83.63% when the sample concentrations were at 1.6, 0.8, 0.4, 0.2 and 0.1 mg/mL. Although Heps also had excellent scavenging activity at high sample concentrations, GHS possessed a superior property and was low cost. Thus, it was worth considering GHS as a promising antioxidant agent in treating ROS-related diseases.

Overall, the result revealed that the GHS exhibited enhanced antioxidant activity, including 98.78% of O_2_•^−^ radical scavenging activity, 91.23% of •OH radical scavenging rate and 66.49% of DPPH radical scavenging capacity at 1.6 mg/mL. It may be because GHS was synthesized by activated cations and anions, which strengthened the bioactivities of the compound. Furthermore, the good yield rate of GHS with a small amount of inorganic ions, can greatly abate preparation cost and furnish economic benefits. So, the findings suggested that GHS has potential to be investigated as a kind of outstanding antioxidant.

## 3. Materials and Methods

### 3.1. Materials

Glucosamine hydrochloride (GAH) was supplied by the MacLin Biochemical Technology Co., Ltd., Shanghai, China. Heparin sodium (Heps) was purchased from the Sigma-Aldrich Chemical Co., Ltd., Shanghai, China. Purified water was supplied by Wa-haha Group Co., Ltd., Shandong, China. Plasma was provided by Sigma-Aldrich Chemical Co., Ltd., Shanghai, China. APTT, PTs and TTs assays reagent were purchased from Sunbiotech Co., Ltd., Shanghai, China. Anhydrous ethanol, trichloroacetic acid, hydrogen peroxide, ethylenediamine tetraacetic acid monosodium ferric salt (EDTA-Fe), 1,1-diphenyl-2-trinitrophenylhydrazine (DPPH), disodium hydrogen phosphate, sodium dihydrogen phosphate, nitro blue tetrazolium (NBT), potassium ferricyanide, phenazine mothosulfate (PMS) trihydroxyaminomethane (Tris), nicotinamideadenine dinucleotid I (NADH), was obtained from Sinopharm Chemical Reagent Co., Ltd., Shanghai, China. These reagents were all analytical grade and were used without further purification.

### 3.2. Analytical Methods

A Jasco-4100 Fourier transform infrared spectrometer (Japan, provided by JASCO Co., Ltd., Shanghai, China), in the range of 4000–400 cm^−1^ with resolution of 4.0 cm^−1^ and at 25 °C in the transmittance mode was used to record all Fourier transform infrared (FTIR) spectra. All samples (about 1 mg) were mixed and grinded with KBr (about 100 mg) fully, and then the ground samples were pressed into pills with a compressor for testing. Meanwhile, all spectra were scanned against a blank KBr pellet background.

The compounds, using 99.9% Deuterium Oxide (D_2_O) as the solvent, were characterized by a nuclear magnetic resonance (NMR) and the ^1^H NMR spectra were analyzed with a Bruker AVIII-500 spectrometer (500 MHz, Switzerland, purchased from Bruker Tech. and Serv. Co., Ltd., Beijing, China).

Trace and ultra-trace elements were detectable by inductively coupled plasma mass spectrometer (ICP-MS) and sodium ions (Na^+^) in samples were determined and analyzed using an Elan DRC II ICP–mass spectrometer instrument (America, supplied by PerkinElmer Co., Ltd., Beijing, China) with a dynamic reaction cell (DRC) technology.

### 3.3. Synthesis of Glucosamine-Heparin Salt

**Scheme 1:**
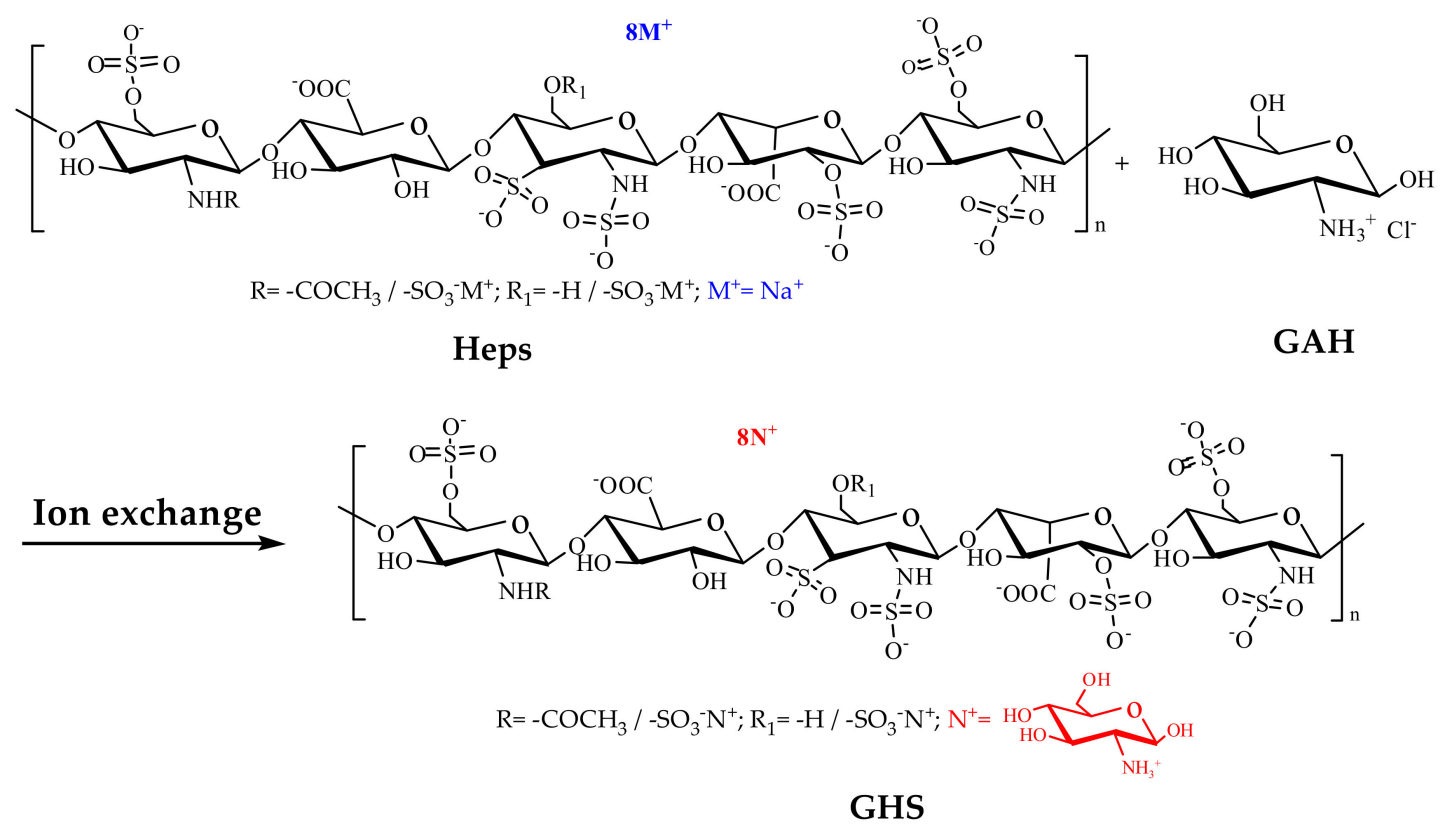
Synthetic route for glucosamine-heparin salt (GHS). In the scheme, the range values of average polymerization degree n are 5–500.

The synthetic route for GHS is showed in Scheme 1. Heparin sodium (1.5 g) was dissolved in 20 mL distilled water stirred for 2 h at 25 °C. Glucosamine hydrochloride (6.0 g) was dissolved in 40 mL distilled water, and the solution was poured slowly into the above heparin sodium solution to react with, stirring continuously for 6 h at 25 °C. Adding the above GAH solution to the heparin sodium solution was repeated three times. After reaction, the product solution was dialyzed with the dialysis bags (Mw = 500) for 24 h. Then, the dialysis products were vacuum-freeze-dried to obtain glucosamine-heparin salt. Yield: 2.79 g, 73.45%.

### 3.4. Anticoagulant Activity Measurement

The anticoagulant activities of all samples were evaluated by determining the activated partial thromboplastin times (APTTs), prothrombin times (PTs) and thrombin times (TTs) in vitro for human plasma, according to the method reported in previous studies with some modifications [23,24,34,35]. Simultaneously, anticoagulant assays were performed using heparin sodium and saline as a reference compound.

#### 3.4.1. Determination of Activated Partial Thromboplastin Times (APTTs)

Solutions of GAH (50 μL), Heps (50 μL), GHS (50 μL) and plasma (50 μL) were preheated and maintained in a water bath at 37 °C. The aqueous solutions of GHS and Heps were added to 0.05 mL of the preheated human plasma and preheated for 60 s at 37 °C. Then, APTT assay reagent (50 μL) was added to the mixture and incubated for 3 min at 37 °C. Thereafter, preheated CaCl_2_ solution (50 μL, 0.025 mol/L) was added and APTTs were recorded. The sample concentrations of 0.75, 1.50 and 2.25 μmol/L were designed in these assays and three repeated tests were carried out for each sample in parallel. All samples were dissolved in saline, and in the control group, normal saline was used.

#### 3.4.2. Determination of Prothrombin Times (PTs)

Mixed solutions of human plasma (50 μL), GAH (50 μL) or Heps (50 μL) or GHS (50 μL) were preheated for 3 min at 37 °C. Then, 0.1 mL of prothrombin reagent preheated for 10~30 min was added to the mixture and incubated for 5 min at 37 °C. Meanwhile, the solidification time was recorded. Each sample was measured at least three times in parallel. All samples were dissolved in saline and the sample concentrations ranged from 0.75~2.25 μmol/L. In the control group, the saline was adopted.

#### 3.4.3. Determination of Thrombin Times (TTs)

GAH (50 μL) or Heps (50 μL) or GHS (50 μL) were preheated and mixed with plasma (50 μL) to maintain constant 37 °C for 3 min. Thereafter, preheated thrombin reagent (0.1 mL) was added and mixed to incubate for 3 min at 37 °C. The TTs were recorded at the same time. The concentration ranges of 0.75~2.25 μmol/L were investigated and three duplicates were measured for each sample in the assays. All samples were dissolved in normal saline, which was used as the negative control.

### 3.5. Antioxidant Assay

The concentrations of these samples, including GAH, Heps and GHS were considered as 10 mg/mL. Their capacities to scavenge hydroxyl radical, DPPH radical and superoxide radical were evaluated according to previous documents with slight modifications [37].

#### 3.5.1. Hydroxyl Radical (•OH) Scavenging Activity Assay

The reaction mixture, containing different concentrations (0.1, 0.2, 0.4, 0.8 and 1.6 mg/mL, 0.5 mL), was incubated with EDTA-Fe^2+^ (0.25 mL), H_2_O_2_ (0.5 mL) and safranine T (0.5 mL) in potassium phosphate buffer (0.5 mL) for 30 min at 37 °C. The absorbance of the mixture was immediately recorded at 520 nm. In the blank, distilled water was used to replace samples. Meanwhile, in the control, distilled water was used to replace samples and H_2_O_2_ was substituted with potassium phosphate solution. Three groups of replicates were conducted for each sample concentration. The hydroxyl radical scavenging capability was calculated by Equation (2):

Scavenging effect (%) = [(A_sample 520 nm_ − A_blank 520 nm_)/(A_control 520 nm_ − A_blank 520 nm_)] × 100 (2)

where A_blank 520 nm_ was the absorbance of the blank and A_control 520 nm_ was the absorbance of the control.

#### 3.5.2. DPPH Radical Scavenging Ability Assay

A deep purple solution of DPPH (0.069 mg/mL, 250 mL) dissolved in absolute ethanol. Sample solutions were attenuated by deionized water at the concentration of 0.1, 0.2, 0.4, 0.8 and 1.6 mg/mL. Afterward, 0.5 mL of diluted solution was thoroughly mixed with 1.0 mL of ethanol solution containing DPPH. In the control, DPPH was substituted with ethanol. Meanwhile, in the blank, samples were substituted with distilled water. Then, the reaction mixture was incubated at 25 °C and protected from light for 20 min. The absorbance at 517 nm of each solution was tested in triplicate to determine the residual amount of DPPH. Three replicates for each sample concentration were measured and the DPPH radical scavenging effect was calculated by a decrease in the absorbance based on Equation (3):

Scavenging effect (%) = [(1 − (A_sample 517 nm_ − A_control 517 nm)_/A_blank 517 nm_] × 100 (3)

where A_sample 517 nm_ was the absorbance of the sample solution, A_control 517 nm_ was the absorbance of the control (ethanol instead of DPPH) and A_blank 517 nm_ was the absorbance of the blank (distilled water instead of the samples).

#### 3.5.3. Superoxide Radical (O_2_•^−^) Scavenging Ability Assay

Briefly, the solutions of Tris-HCl (1.9382 mg/mL, 500 mL) buffer, nitro blue tetrazolium (NBT, 0.245 mg/mL, 100 mL), nicotinamide adenine dinucleotide (NADH, 0.366 mg/mL, 100 mL) and phenazine methosulfate (PMS, 0.045 mg/mL, 100 mL) were prepared to start. In each tube, 0.75 mL of sample solution with different concentrations (0.1, 0.2, 0.4, 0.8 and 1.6 mg/mL) was mixed with 0.25 mL NADH, 0.25 mL NBT and 0.25 mL PMS solution to the mixture. The reaction mixture was incubated for 10 min at room temperature and the absorbance was read at 560 nm against a blank. Samples were substituted with distilled water in the blank. Meanwhile, in the control, NADH was substituted with distilled water. Three replicates for each sample concentration were measured and the capability of scavenging superoxide radical was calculated using Equation (4):

Scavenging effect (%) = [(1 − (A_sample 560 nm_ − A_control 560 nm_)/A_blank 560 nm_] × 100 (4)

where A_sample 560 nm_ was the absorbance of sample solution, A_control 560 nm_ was the absorbance of the control (deionized water instead of sample solution) and A_blank 560 nm_ was the absorbance of the blank (sample solution with Tris-HCl buffer instead of NBT solution).

### 3.6. Statistical Analysis

Each experience was performed three times, and all data were expressed as means ± SD. Data were analyzed by an analysis of variance (*p* < 0.05) to guarantee statistical significance and the one-way analysis of variance (ANOVA) was used to evaluate all experimental data. The results were processed by the computer program, Origin.

## 4. Conclusions

In this paper, glucosamine-heparin salt was prepared by ion exchange method using negatively charged heparin sodium and positively charged glucosamine hydrochloride. The structural information of GHS was thoroughly characterized by FTIR, ^1^H NMR and ICP-MS. In addition, its anticoagulant potencies and antioxidant properties were evaluated. Results revealed that both antioxidant activity and anticoagulant ability of GHS had been enhanced. The superoxide radical scavenging rate achieved 98.78%, the DPPH radical scavenging ability was 66.49% and the hydroxyl radical scavenging capacity reached 91.23% at 1.6 mg/mL. At the same time, anticoagulant ability results demonstrated that the strengthened anticoagulant property of GHS was achieved with emerging prolonged APTTs, PTs and TTs compared to that of heparin sodium. It is worth noting that introducing cationic glucosamine residues could promote anticoagulant activity of GHS even though its anticoagulant activity was improved slightly. Nevertheless, the prepared GHS possessed inviting preponderances, including low preparation cost, a comparatively high DS and good yield rate. Furthermore, GHS compound composed of two kinds of activated synergistic actions, contained small amount of inorganic ions and could abate the risk of electrolyte disorder in vivo. Thereby, the investigations suggest that GHS has tremendous potential to serve as a kind of outstanding anticoagulant and antioxidant in the future with broad application prospects and momentous economic significance.

## Figures and Tables

**Table 1:** Determination of sodium ion (Na^+^) of Heps and GHS.

Sample ^a^	Concentration Mean (Na^+^) ng/mL
Heps	2.4 × 10^5^ ± 1.10
GHS	4520 ± 0.50

^a^ Concentration of Heps and GHS was 1.0 × 10^−3^ g/mL.

**Table 2:** Anticoagulant activities of GAH, Heps and GHS measured by APTT, PT and TT assays.

Sample	APTT (s) ^a^			PT (s) ^a^			TT (s) ^a^		
	0.75 μmol/L P ^b^	1.50 μmol/L P ^b^	2.25 μmol/L P ^b^	0.75 μmol/L P ^b^	1.50 μmol/L P ^b^	2.25 μmol/L P ^b^	0.75 μmol/L P ^b^	1.50 μmol/L P ^b^	2.25 μmol/L P ^b^
Saline	102.88 ± 4.30			26.50 ± 0.41			18.22 ± 0.34		
GAH	103.23 ± 2.55			26.37 ± 0.66			17.89 ± 0.49		
Heps	153.10 ± 17.14	181.97 ± 2.60	≥300	26.99 ± 1.10	28.37 ± 0.29	33.93 ± 0.45	19.63 ± 0.21	25.50 ± 4.45	≥100
GHS	180.03 ± 6.02	≥300	≥300	28.30 ± 0.62	30.67 ± 0.77	≥40	22.90 ± 0.36	28.70 ± 1.31	≥100

^a^ Results are expressed as mean ± S.D. (*n* = 3). ^b^ Plasma.

## Data Availability

All data were contained in the manuscript.

## Sample Availability

Samples of the compounds are available from the authors.

## Appendix A

The FTIR spectra of GAH, Heps and GHS were displayed in Figure A1. No new characteristic absorptions appeared when comparing the FTIR spectra of the new GHS derivative and that of Heps.
